# Predictors of the Need to Use Medications in the Management of Neonatal Hypoglycemia

**DOI:** 10.7759/cureus.32197

**Published:** 2022-12-05

**Authors:** Samira Al Housni, Khadija Al Ghaithi, Sathiya P. Murthi, Hussain Alsaffar, Hilal Al Mandhari

**Affiliations:** 1 Pediatrics Department, Oman Medical Specialty Board, Muscat, OMN; 2 Pediatrics Department, Sohar Hospital, Sohar, OMN; 3 Statistics, Oman Medical Specialty Board, Muscat, OMN; 4 Child Health Department - Pediatric Endocrinology Unit, Sultan Qaboos University Hospital, Muscat, OMN; 5 Neonatology/Child Health Department, Sultan Qaboos University Hospital, Muscat, OMN

**Keywords:** medication use, therapy, risk factors, newborn, hypoglycemia

## Abstract

Background and objective

Neonatal hypoglycemia (NH) is one of the most common causes of admission to the neonatal intensive care unit (NICU). Persistent NH despite adequate feeding and intravenous dextrose may often require medications to maintain normal blood glucose levels (BGL). Several medications are used in the management of persistent NH, such as glucagon, diazoxide, and octreotide. In this study, we aimed to determine the factors that predict the need for medications to treat persistent NH.

Methods

This was a retrospective cohort study conducted at the Sultan Qaboos University Hospital (SQUH), Muscat, Oman. Infants admitted to the NICU between 2015 and 2019 with hypoglycemia (capillary blood glucose <2.6 mmol/l) were eligible to be included in the study. A prespecified dataset was collected from electronic patient records, including birth weight (BW), APGAR scores, gestational age, BGL, maternal risk factors such as diabetes mellitus (DM), hypertension, or antenatal use of medications, and the NICU management during admission. Data analysis was performed using SPSS Statistics for Windows, version 27.0 (IBM Corp., Armonk, NY).

Results

A total of 89 neonates were admitted due to NH during the study period. Of them, 10 (11.2%) patients had received medication (diazoxide). Use of medication for persistent NH was significantly associated with maternal gestational diabetes/diabetes mellitus (GDM/DM) status (p=0.041), higher BW (p=0.001), and large for gestational age [LGA (defined as BW >90th percentile)] (p=0.014), severe hypoglycemia (mean glucose level of 1-1.5 mmol/l) at two hours of life and at admission, and elevated maximum glucose infusion rate (GIR). GIR for the medication-requiring cohort was 12.95 mg/kg/min and that for the non-medication-requiring cohort was 6.77 mg/kg/min (p<0.001).

Conclusion

Based on our findings, the need for using certain medications to treat persistent NH, such as diazoxide in neonates admitted with NH, can be predicted by factors such as maternal GDM/DM status, BW >90th percentile, very low BGL at two hours of age and on admission, and elevated GIR. Elevated maximum GIR was a leading indicator for using medications in the treatment of NH.

## Introduction

Hypoglycemia is a common cause of admission to the neonatal intensive care unit (NICU) [1]. The incidence of neonatal hypoglycemia (NH) is variable, largely due to inconsistent definitions and cut-offs as well as varying methods of measuring blood glucose concentrations [2,3]. The World Health Organization (WHO) defines hypoglycemia as “blood glucose level (BGL) of less than 45 mg/dL (2.5 mmol/L) [4]. In our practice at the Sultan Qaboos University Hospital (SQUH) NICU, NH is defined as blood sugar of less than 2.6 mmol/L (47 mg/dL) within the first 48 hours of birth and less than 3.3 mmol/L (60 mg/dL) thereafter.

Antenatally, the fetal brain receives glucose from maternal plasma through the placenta. However, with the separation of the placenta soon after birth, there is a physiological decrease in the blood glucose of the newborn, with a nadir reached at around one to two hours of life [5]. Therefore, blood glucose will vary and it depends on multiple factors including birth weight (BW), gestational age, feeding status, availability of energy sources, body stores, as well as presence or absence of disease [6,7]. Hypoglycemia is more common in certain high-risk groups such as sick infants, e.g., asphyxiated, septic, and hypothermic infants as well as preterm, small, and large for gestational age [LGA (defined as BW >90th percentile)] infants, and infants of diabetic mothers (IDM) [1].

The symptoms and signs of NH are nonspecific and reflect the responses of the nervous system to glucose deprivation. Most of the neonates are asymptomatic while others show symptoms such as poor suck or feeding, weak or high-pitched crying, lethargy, hypotonia, or seizures. Furthermore, some others may develop autonomic symptoms including jitteriness, tremors, sweating, irritability, and tachypnoea [7].

NH is classified into transient and persistent types, mainly based on the duration of hypoglycemia. Transient NH is defined as low glucose concentration during the first 48 hours of life. However, persistent NH is defined as the persistence of low plasma glucose concentrations beyond the first 48 hours of life [7,8]. There are several etiologies pertaining to this classification. Hyperinsulinism is the most common cause of persistent NH. It is characterized by inappropriate secretion of insulin in the presence of low plasma glucose. It can be transient or persistent. Other less common causes of persistent hypoglycemia are counterregulatory hormone deficiencies, defects in gluconeogenesis and glycogenolysis, galactosemia, and other metabolic disorders [9].

When conservative measures fail to correct the NH, medications would be needed, such as glucagon, diazoxide, octreotide, sirolimus, and nifedipine [10-16]. Diazoxide is the first-line drug approved by the US Food and Drug Administration (FDA) [1,17]. However, diazoxide use has been associated with certain adverse effects, such as the development of fluid retention and, less commonly, respiratory decompensation and pulmonary hypertension [18-20]. At SQUH, diazoxide is the first-line medication for treating persistent NH.

This study’s main objective is to determine the risk factors that predict the need to use medical therapy (diazoxide) for persistent hypoglycemia in neonates as timely interventions are crucial to prevent long-term sequelae.

## Materials and methods

Study design and setting

This was a retrospective cohort study conducted at the Sultan Qaboos University Hospital (SQUH), Muscat, Oman. SQUH is a tertiary-care level and academic medical center. The NICU at SQUH is a level III facility with a 24-cot capacity. SQUH is a maternity center with an average of about 5000 deliveries per year. The study obtained ethical approval from the institutional Medical Research Ethical Committee (MREC #2648).

Infants born in SQUH are routinely screened for hypoglycemia if they are deemed to be at risk, e.g., preterm infants, infants with low BW and intrauterine growth restriction (IUGR), IDM, and LGA infants. Blood glucose is checked at two hours of age after giving their initial feed within the first hour of life. If blood sugar is <2.6 mmol/L (47 mg/dL), a top-up feed is given and blood sugar is checked again after one hour. Failure to maintain a blood sugar level >2.6 mmo/L (47 mg/dL) after a top-up feed is an indication for admission to the NICU for the administration of intravenous fluids (IVFs). Neonates admitted with hypoglycemia are initially started on dextrose at a glucose infusion rate (GIR) of 5.5 mg/Kg/min. Infants are allowed to be fed orally on demand. Blood sugar is monitored every three hours pre-feeds. If hypoglycemia persists, GIR is gradually increased. Medications are often considered in consultation with the pediatric endocrinology team in neonates with persistent hypoglycemia and intravenous GIR of 10 mg/Kg/min or difficulty in weaning off IV dextrose.

Study population

The study population included neonates who were born at SQUH between 2015 and 2019 and were admitted to NICU with hypoglycemia [defined as blood glucose <2.6 mmol/L (47 mg/dL)].

Data collection

After obtaining ethical approval for the study proposal, patients' electronic charts were reviewed and the following data were collected from electronic patient records: maternal information including age, antenatal complications, antenatal use of steroids or beta-blockers, and spontaneous onset of preterm labor; birth information including gestational age, APGAR scores, gender, the year of birth, mode of delivery, BW and head circumference and their percentiles on growth charts, admission temperature, blood group, and initial blood glucose; neonatal morbidities including prematurity, IUGR, whether IDM, sepsis, or metabolic diseases; information on the management of hypoglycemia including feeding regimen, IVFs, use of medications, calculated maximum GIR, duration of treatment, and duration of hospital stay. 

Statistical analysis

For the purposes of analysis, the study population was divided into two cohorts: “no medication” and “medication” cohorts. SPSS Statistics for Windows, version 27.0 (IBM Corp., Armonk, NY) was used for the data analysis. Continuous variables were expressed as mean ± standard deviation (SD) or median [interquartile range (IQR)] and categorical variables were presented as numbers and percentages. The two cohorts were compared in terms of their baseline characteristics. The Chi-square test was used to test the significance of the association between categorical variables, and the non-parametric Mann-Whitney U test was used to test the significance of the difference between the means of the two groups. Regression analysis was performed on factors that were significantly associated with the use of medication; odds ratio (OR) and 95% confidence intervals (CI) were calculated to determine predictors of the need for medications in the management of NH. A p-value ≤0.05 was considered statistically significant.

## Results

During the study period, 89 newborn infants were admitted to NICU with hypoglycemia [defined as a blood glucose of less than 2.6 mmol/L (47 mg/dL)] within the first 48 hours of birth; 10 of them (11.2%) received medication (all received diazoxide) during their hospitalization in the NICU for persistent hypoglycemia. The comparison of demographics and other variables is presented in Table 1. Significantly, the mothers of 90% of infants in the medication cohort had GDM/DM during pregnancy (p=0.041). However, none of these mothers were on insulin as compared to 31.7% of those in the no-medication cohort. No significant difference was observed in maternal HbA1C levels and the use of beta-blockers during pregnancy between the groups (Table 1). Similarly, no significant difference was observed in ratios of term and preterm gestational ages. Also, no significant differences were observed between the two cohorts in the mode of delivery, APGAR scores at one minute and five minutes, gender, and admission temperature (Table 1).

**Table 1:** Baseline characteristics and risk factors of study cohorts *Mann-Whitney test; ^a^variable expressed as mean ±SD; ^b^variable expressed as median (IQR) P-value ≤0.05 is significant BW: birth weight; DM: diabetes mellitus; GA: gestational age; GDM: gestational diabetes mellitus; GH: growth hormone; GIR: glucose infusion rate; IDM: infant of diabetic mother; IQR: interquartile range; SD: standard deviation; SVD: spontaneous vaginal delivery

Variables	No medication (n=79)		Medication (n=10)		P-value
	N	%	N	%	
Maternal GDM/DM	43	54.4	9	90.0	0.041
GA (weeks)					0.208
Term (≥37 weeks)	33	41.8	7	70.0	
Late preterm (34–<36 weeks)	44	55.7	3	30.0	
Very preterm (28–33 weeks)	2	2.5	-	­-	
GDM/DM treatment					0.038
Diet	16	39.0	7	77.8	
Metformin	9	22.0	2	22.2	
Insulin	13	31.7	-	-	
Combined	3	7.3	-	-	
HbA1c^a^	6.89 (1.87)		5.57 (0.99)		0.185
Maternal beta blockers	6	7.6	2	20.0	0.220
BW^a^	2.49 (0.78)		3.36 (0.85)		0.001
BW percentile					0.014
<10 percentile	34	43.0	2	20.0	
10-90 percentile	37	46.8	3	30.0	
>90 percentile	8	10.1	5	50.0	
BW					0.502
Normal (10–90th percentile)	37	46.8	3	30.0	
Abnormal (<10th or >90th percentile)	42	53.2	7	70.0	
Gender (male)	46	58.2	6	60.0	1.000
Mode of delivery (SVD)	47	60.3	7	70.0	0.832
APGAR score at 1 minute^a^	8.13 (1.46)		7.20 (2.74)		0.159
APGAR score at 5 minutes^a^	9.43 (0.75)		8.90 (1.20)		0.102
Glucose at 2 hours^a^ (mmol/L)	2.18 (1.07)		1.13 (0.58)		0.002
Glucose at admission^a^ (mmol/L)	2.27 (0.98)		1.36 (0.76)		0.006
Admission temperature^a^ (NICU)	36.0 (1.32)		36.44 (0.30)		0.296
Max. GIR^a^ (mg/Kg/min)	6.74 (2.69)		12.95 (1.78)		<0.001
Length of stay^b^ (days)	7 (5, 11)		9 (7.75, 24.5)		0.013*
Critical sample done	6	7.6	5	50.0	0.002
Insulin^b^ (mIU/L)	4.75 (2.25, 19.7)		13 (1.3, 13)		0.857*
Cortisol^a^ (nmol/L)	476.80 (150.12)		404 (97.92)		0.488
GH^a^ (mIU/L)	62.06 (28.19)		61.52 (23.23)		0.979

The mean BW of the medication cohort was 3.36 ±2.49 Kg, which was significantly higher than that of the no-medication cohort (2.49 ±0.78 kg, p=0.001). Infants exposed to medications were more likely to be LGA (p=0.014), infants of GDM/DM mothers (90%) (p=0.041), had lower blood glucose at two hours of life and on admission, and had higher maximum GIR (mean maximum GIR was 12.95 ±1.78 mg/kg/min) (p<0.001) compared with the no-medication cohort.

A critical sample was performed in 50% of the medication cohort (p=0.002). The median insulin level was higher in the medication cohort; however, the difference was not statistically significant. Mean cortisol and growth hormone (GH) levels were slightly lower in the medication cohort, but this again was not statistically significant. The median length of stay for infants in the medication cohort was nine days, which was significantly longer than that of the no-medication cohort (seven days) (p=0.013). There was no documented adverse effect of diazoxide use among neonates studied. 

A logistic regression was performed to determine the effects of risk factors on the likelihood that patients received medication (diazoxide). After adjusting for factors (GDM, APGAR, BW percentile, blood sugar at two hours of life, and maximum GIR required), only the maximum GIR was found to be an independent predictor. Increasing maximum GIR was statistically associated with an increased likelihood of use of medication for hypoglycemia (OR: 3.10, 95% CI: 1.22-7.87, p=0.018) (Table 2). GDM status of the mother or being IDM was almost close to being a significant predictor of medication need (OR: 0.005, 95% CI: 0.01-1.69, p=0.075).

**Table 2:** Logistic regression analysis results P-value ≤0.05 is significant BW: birth weight; GDM: gestational diabetes mellitus; GIR: glucose infusion rate

Variables	B	P-value	OR	95% CI
GDM	-5.256	0.075	0.005	0.01–1.69
BW percentile (reference: 10–90th percentile)				
<10th percentile	-0.768	0.634	0.464	0.02–10.98
>90th percentile	1.993	0.271	7.339	0.21–255.43
APGAR score at 1 minute	-0.088	0.858	0.916	0.35–2.41
Blood glucose at 2 hours of life	-1.305	0.198	0.271	0.04–1.98
Max. GIR required	1.104	0.021	3.015	1.18–7.70

## Discussion

Hypoglycemia remains one of the most common biochemical disorders in neonates. However, there is still no consensus on its definition, diagnosis, and treatment [9]. Various medications are used in treatment protocols for persistent NH, such as glucagon, diazoxide, octreotide, sirolimus, and nifedipine [10-14]. Diazoxide is the first-line medication used to treat persistent NH in neonates admitted to SQUH’s NICU, where this study was conducted. This retrospective study was conducted to determine factors that may predict the need for medical therapy as part of the treatment of NH. Based on our findings, medication use is significantly more common in infants who are LGA, infants born to diabetic mothers, those with very low blood glucose at two hours of age and at admission, and those with a requirement of high intravenous GIR. Interestingly, on binary regression analysis, high maximum GIR was found to be the only significant predictor of medication use (diazoxide in this study).

To our knowledge, this is the first study to analyze risk factors and predictors of the need to use medications to treat NH. All infants who received medications (diazoxide) had a maximum IV GIR of >10 mg/kg/min (mean of about 12 mg/kg/min). Several previous studies have focused on investigating the risk factors for NH, e.g., prematurity, low BW, IUGR, LGA, maternal DM, and perinatal asphyxia [21-27]. In the study by Harris et al. in 2012, 51% of at-risk infants developed hypoglycemia (<2.6 mmol/L) and most episodes of hypoglycemia (81%) occurred in the first 24 hours of birth [23].

The need for medication for the management of NH was also associated with maternal GDM/DM or being IDM and higher BW. Routine glucose testing is indicated in LGA newborn infants of nondiabetic mothers as we found it to be significantly associated with hypoglycemia and the need for diazoxide.

There are a few strengths and limitations of this study that are worth discussing. The strength mainly lies in the fact that this is the first study to investigate the predictors of the need to use medications in the management of persistent NH. The study was done over a period of five years. Limitations of this study are related to its retrospective cohort study design, which limited its ability to establish causality, and that it represents a single-center experience and relatively small sample size. We recommend larger, prospective, and multicenter studies to gain deeper insights into factors that may help predict the need for medical treatment for persistent NH.

## Conclusions

In this single-center study, the need for medical therapy such as diazoxide in neonates admitted with NH in NICU was associated with maternal GDM/DM status, higher BW and BW >90th percentile, very low BGL at two hours of age and on admission, and high GIR. Elevated GIR (>10-12 mg/kg/min) was the only factor that predicted the need for medical therapy. We recommend larger, prospective, and multicenter studies to further investigate the predictors of the need for medications to treat NH.

## References

[1] Kallem VR, Pandita A, Gupta G (2017). Hypoglycemia: when to treat?. Clin Med Insights Pediatr.

[2] Bülbül A, Bahar S, Uslu S, Sözeri Ş, Bülbül L, Baş EK, Ünal ET (2019). Risk factor assessment and the incidence of neonatal hypoglycemia in the postnatal period. Sisli Etfal Hastan Tip Bul.

[3] Hosagasi NH, Aydin M, Zenciroglu A, Ustun N, Beken S (2018). Incidence of hypoglycemia in newborns at risk and an audit of the 2011 American academy of pediatrics guideline for hypoglycemia. Pediatr Neonatol.

[4] Adamkin DH (2017). Neonatal hypoglycemia. Semin Fetal Neonatal Med.

[5] Arya VB, Senniappan S, Guemes M, Hussain K (2014). Neonatal hypoglycemia. Indian J Pediatr.

[6] Hoseth E, Joergensen A, Ebbesen F, Moeller M (2000). Blood glucose levels in a population of healthy, breast fed, term infants of appropriate size for gestational age. Arch Dis Child Fetal Neonatal Ed.

[7] Cornblath M, Hawdon JM, Williams AF, Aynsley-Green A, Ward-Platt MP, Schwartz R, Kalhan SC (2000). Controversies regarding definition of neonatal hypoglycemia: suggested operational thresholds. Pediatrics.

[8] Thornton PS, Stanley CA, De Leon DD (2015). Recommendations from the Pediatric Endocrine Society for evaluation and management of persistent hypoglycemia in neonates, infants, and children. J Pediatr.

[9] Stanley CA, Rozance PJ, Thornton PS (2015). Re-evaluating "transitional neonatal hypoglycemia": mechanism and implications for management. J Pediatr.

[10] Volpe JJ, Inder T, Darras B, de Vries L, du Plessis A, Neil J, Perlman J (2017). Glucose. Volpe's Neurology of the Newborn.

[11] Cornblath M, Schwartz R (1999). Outcome of neonatal hypoglycaemia. Complete data are needed. BMJ.

[12] Chandran S, Rajadurai V, Alim A, Hussain K (2015). Current perspectives on neonatal hypoglycemia, its management, and cerebral injury risk. Res Rep Neonatol.

[13] Thornton PS, Alter CA, Katz LE, Baker L, Stanley CA (1993). Short- and long-term use of octreotide in the treatment of congenital hyperinsulinism. J Pediatr.

[14] Demirbilek H, Shah P, Arya VB, Hinchey L, Flanagan SE, Ellard S, Hussain K (2014). Long-term follow-up of children with congenital hyperinsulinism on octreotide therapy. J Clin Endocrinol Metab.

[15] Baş F, Darendeliler F, Demirkol D, Bundak R, Saka N, Günöz H (1999). Successful therapy with calcium channel blocker (nifedipine) in persistent neonatal hyperinsulinemic hypoglycemia of infancy. J Pediatr Endocrinol Metab.

[16] Senniappan S, Alexandrescu S, Tatevian N (2014). Sirolimus therapy in infants with severe hyperinsulinemic hypoglycemia. N Engl J Med.

[17] (2021). National Library of Medicine. Dailey Med. Package insert: Proglycem-diazoxide suspension. https://dailymed.nlm.nih.gov/dailymed/drugInfo.cfm?setid=b16c7832-2fd9-49af-b923-1dc0d91fd6e2.

[18] Arnon S, Lagerev E, Herzlich J, Eliakim A, Litmanovitz I (2014). Diazoxide treatment for persistent hypoglycemia in a small for gestational age preterm infant with adequate low insulin levels. Case Rep Perinat Med.

[19] Brar PC, Heksch R, Cossen K (2020). Management and appropriate use of diazoxide in infants and children with hyperinsulinism. J Clin Endocrinol Metab.

[20] Desai J, Key L, Swindall A, Gaston K, Talati AJ (2021). The danger of diazoxide in the neonatal intensive care unit. Ther Adv Drug Saf.

[21] Cummings CT, Ritter V, LeBlanc S, Sutton AG (2022). Evaluation of risk factors and approach to screening for asymptomatic neonatal hypoglycemia. Neonatology.

[22] Bromiker R, Perry A, Kasirer Y, Einav S, Klinger G, Levy-Khademi F (2019). Early neonatal hypoglycemia: incidence of and risk factors. A cohort study using universal point of care screening. J Matern Fetal Neonatal Med.

[23] Harris DL, Weston PJ, Harding JE (2012). Incidence of neonatal hypoglycemia in babies identified as at risk. J Pediatr.

[24] Mitchell NA, Grimbly C, Rosolowsky ET, O'Reilly M, Yaskina M, Cheung PY, Schmölzer GM (2020). Incidence and risk factors for hypoglycemia during fetal-to-neonatal transition in premature infants. Front Pediatr.

[25] Stomnaroska O, Petkovska E, Jancevska S, Danilovski D (2017). Neonatal hypoglycemia: risk factors and outcomes. Pril (Makedon Akad Nauk Umet Odd Med Nauki).

[26] Turner D, Monthé-Drèze C, Cherkerzian S, Gregory K, Sen S (2019). Maternal obesity and cesarean section delivery: additional risk factors for neonatal hypoglycemia?. J Perinatol.

[27] Zhou W, Yu J, Wu Y, Zhang H (2015). Hypoglycemia incidence and risk factors assessment in hospitalized neonates. J Matern Fetal Neonatal Med.

